# Erratum to: The influence of N-acetyl-L-cysteine infusion on cytokine levels and gastric intramucosal pH during severe sepsis

**DOI:** 10.1186/s13054-016-1572-2

**Published:** 2016-12-19

**Authors:** Sayim Emet, Dilek Memis, Zafer Pamukcu

**Affiliations:** Department of Anaesthesiology and Reaniamation, Medical Faculty, Trakya University, Edirne, Turkey

## Erratum

Following publication of the original version of our article [1] in *Critical Care*, it was brought to our attention that there was an error in Table 3. Some of the values are incorrect, including arterial oxygen saturation variables and a standard deviation in arterial carbon dioxide tension (mmHg) control group (24 h after N-acetyl-L-cysteine infusion). The corrected table is presented in this erratum (Table 3). This error does not affect the results or conclusions of the study.

**Table 3:** Hemodynamic, oxygen and temperature variables

Variable	Baseline	Immediately after NAC infusion	24 h after NAC infusion	48 h after NAC infusion
Heart rate (beats/min)				
NAC	110.88 ± 25.9	113.00 ± 25.4	112.74 ± 22.4	108.70 ± 22.2
Control	104.84 ± 18.2	105.19 ± 23.5	105.23 ± 23.5	103.15 ± 22.7
Mean arterial pressure (mmHg))				
NAC	90.85 ± 16.67	88.92 ± 13.01	86.07 ± 13.21	85.96 ± 13.41
Control	91.26 ± 18.25	83.80 ± 14.77	85.42 ± 13.42	83.80 ± 11.31
Arterial pH				
NAC	7.34 ± 0.10	7.41 ± 0.07	7.38 ± 0.10	7.39 ± 0.06
Control	7.35 ± 0.09	7.38 ± 0.07	7.39 ± 0.08	7.36 ± 0.08
Arterial carbon dioxide tension (mmHg)				
NAC	35.88 ± 12.7	34.62 ± 18.8	35,00 ± 14.2	38.54 ± 151
Control	31.69 ± 20.1	38.56 ± 20.2	36,46 ± 7.1	34.33 ± 21.6
Arterial oxygen tension/fractional insired oxygen ratio (mmHg)				
NAC	172 ± 88	176 ± 76	178 ± 81	179 ± 126
Control	174 ± 76	177 ± 65	181 ± 70	179 ± 68
Arterial oxygen saturation (%)				
NAC	95.4 ± 3.6	95.5 ± 3.1	94.7 ± 3.6	92.6 ± 5.3
Control	94.6 ± 3.6	94.1 ± 3.3	93.8 ± 3.1	94.8 ± 3.1
Temperature (°C)				
NAC	36.8 ± 0.75	37 ± 0.78	36.8 ± 0.75	36.9 ± 0.48
Control	36.6 ± 0.57	36.8 ± 0.45	36.6 ± 0.57	36.8 ± 0.53

Data are expressed as mean ± standard deviation. We identified no statistically significant differences between groups. NAC,N-acetyl-L-cysteine
